# The rediscovery of the putative ant social parasite *Manica parasitica* syn. nov. (Hymenoptera: Formicidae) reveals an unexpected endoparasite syndrome

**DOI:** 10.1098/rsbl.2023.0399

**Published:** 2023-12-20

**Authors:** Matthew Prebus, Boyko B. Georgiev, Thomas van de Kamp, Elias Hamann, Iyla Baker, Christian Rabeling

**Affiliations:** ^1^ Social Insect Research Group, School of Life Sciences, Arizona State University, 550 E Orange St., Tempe, AZ 85281, USA; ^2^ Department of Integrative Taxonomy of Insects, Institute of Biology, University of Hohenheim, Garbenstraße 30, 70599, Stuttgart, Germany; ^3^ KomBioTa – Center for Biodiversity and Integrative Taxonomy Research, University of Hohenheim and State Museum of Natural History Stuttgart, Germany; ^4^ Institute of Biodiversity and Ecosystem Research, Bulgarian Academy of Sciences, 2 Gagarin Street, 1113 Sofia, Bulgaria; ^5^ Institute for Photon Science and Synchrotron Radiation (IPS), Karlsruhe Institute of Technology (KIT), Hermann-von-Helmholtz-Platz 1, 76344 Eggenstein-Leopoldshafen, Germany; ^6^ Laboratory for Applications of Synchrotron Radiation (LAS), Karlsruhe Institute of Technology (KIT), Kaiserstraße 12, 76131 Karlsruhe, Germany; ^7^ Department of Neurobiology, Northwestern University, 633 Clark St, Evanston, IL 60208, USA

**Keywords:** Cestoda, Formicidae, inquiline syndrome, integrative taxonomy‌, mermithogenic syndrome, tapeworm, *Raillietina*

## Abstract

Parasitism is ubiquitous across the tree of life, and parasites comprise approximately half of all animal species. Social insect colonies attract many pathogens, endo- and ectoparasites, and are exploited by social parasites, which usurp the social environment of their hosts for survival and reproduction. Exploitation by parasites and pathogens versus social parasites may cause similar behavioural and morphological modifications of the host. Ants possess two overlapping syndromes: the endo- and social parasite syndromes. We rediscovered two populations of the putative social parasite *Manica parasitica* in the Sierra Nevada, and tested the hypothesis that *M. parasitica* is an independently evolving social parasite*.* We evaluated traits used to discriminate *M. parasitica* from its host *Manica bradleyi,* and examined the morphology of *M. parasitica* in the context of ant parasitic syndromes. We find that *M. parasitica* is not a social parasite. Instead, *M. parasitica* represents cestode-infected *M. bradleyi*. We propose that *M. parasitica* should be regarded as a junior synonym of *M. bradleyi*. Our results emphasize that an integrative approach is essential for unravelling the complex life histories of social insects and their symbionts.

## 1. Introduction

Parasitism is characterized by one organism exploiting another organism by feeding on it, showing adaptations to it and harming it [1]. Parasitism is ubiquitous across the tree of life, and parasites can drive biological diversification [2]. Approximately half of all animal species are parasites, and parasitism has evolved more than 200 times in animals [3,4]. Social insects provide resources for parasites and ideal conditions for parasite transmission because they occur abundantly in terrestrial ecosystems, and live in large, densely packed colonies which often persist for multiple years [5,6]. Accordingly, colonies of social insects are exploited by many pathogens, endo- and ectoparasites, as well as parasitoids [5].

Ant colonies in particular are inhabited by a menagerie of guests, the so-called myrmecophiles, which are distantly related invertebrates that steal resources or prey on the ants' brood [7]. By contrast, social parasitism is a phenomenon that is common *between* ant species. Here, a parasitic species exploits the social behaviour of another, often closely-related free-living species to survive and reproduce [8]. Of the approximately 14 000 recognized ant species [9], more than 400 social parasite species are known, which evolved at least 91 times independently across the ant tree of life [10,11]. Furthermore, ants are hosts of many endoparasites, including nematodes, cestodes, trematodes, fungi, bacteria and viruses [5,12,13].

Among these host–parasite pairs, strong selection acts on both partners, generating morphological, physiological and behavioural syndromes, which indicate host–parasite co-evolution [13,14]. One well-studied ant endoparasite syndrome is the mermithogenic syndrome, where mermithid nematodes develop for parts of their life cycles inside ant hosts [15]. Upon maturity, the nematode alters the host's behaviour, and the infected ant drowns itself, releasing the parasite [16]. Mermithid infections can cause modifications to the host's morphology, including mosaic phenotypes in females and intercastes [17]. Some morphological alterations are so extreme that parasitized individuals were erroneously described as new species, causing taxonomic confusion [15,18,19]. Another established syndrome in ants is the social parasite syndrome. Here, the morphology, behaviour, and reproductive patterns of ant social parasites are adapted to the parasitic life history. For example, queens of dulotic species kill the host queen during colony founding, taking over the host colony; the worker caste bears saber-shaped mandibles specialized for assaulting host colonies and stealing their pupae [20]. In contrast, inquiline parasites often coexist with the host queen and are characterized by reduced size, loss of the worker caste, reduced mouthparts and sib-mating inside the nest [14,21–24].

**Figure 1.**
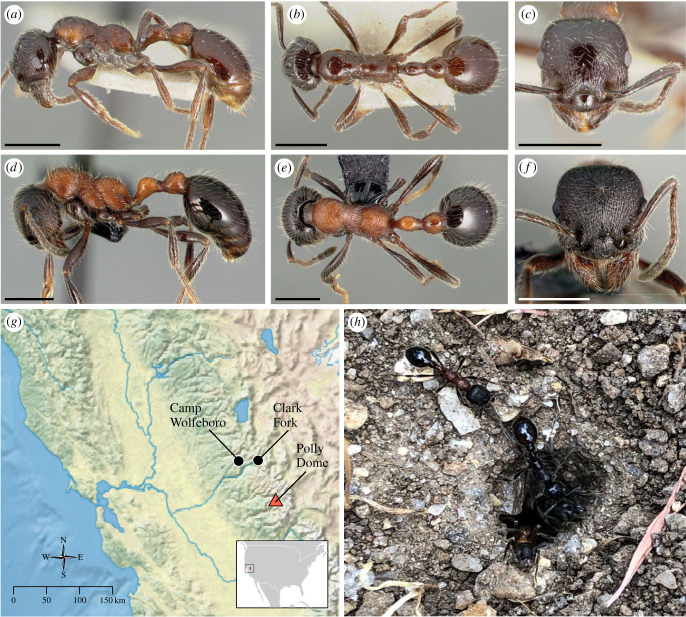
Morphology and geographical distribution of *Manica parasitica* and *Manica bradleyi*. *Manica parasitica* worker (paratype originally designated as cotype; unique specimen identifier CASENT0005974; photographer April Nobile, from www.antweb.org) in (*a*) profile (*b*) dorsal and (*c*) full-face view; *M. bradleyi* worker (CASENT0005697; photographer April Nobile, from www.antweb.org) in (*d*) profile (*e*) dorsal and (*f*) full-face view; (*g*) map of collection localities used in this study (black circles) and type locality of *M. parasitica* (red triangle); (*h*) photograph of *M. parasitica* entering a nest of *M. bradleyi* taken at Clark Fork, CA.

The endoparasite and social parasite syndromes present overlapping shifts in morphology and behaviour (table 1). Therefore, disentangling these syndromes is challenging when presented with morphology alone. Here, we examine the biology of the putative social parasite *Manica parasitica* (Creighton, 1934). *Manica parasitica* (figure 1*a–c*) was first collected in the nest of *Manica bradleyi* (Wheeler, 1909) (figure 1*d–f*) in Yosemite National Park in California [25]. Only two additional collections have been documented, both in the Sierra Nevada. Creighton classified *M. parasitica* as a social parasite of *M. bradleyi* due to its co-nesting behaviour and morphology, which overlaps with the social parasite syndrome, such as shiny integument, smaller workers, reduced propodeum and a lower petiole height compared to *M. bradleyi* [25]. Creighton [25] assumed temporary social parasitism because putative parasite and host workers occupy the same nest, and lack of mandibular specialization did not indicate dulosis. Later, Wheeler & Wheeler [26] noted *M. parasitica* ambulated differently than *M. bradleyi*, but their conclusion was based upon a single worker, and they could not rule out that it was injured [26]. We revisited two historical *M. parasitica* collecting localities and excavated nests containing *M. bradleyi* and *M. parasitica.* To determine the relationship between the two, we critically re-evaluate the status of *M. parasitica* as a symbiont and as a species using multiple lines of evidence.

**Table 1.** Traits of the social parasite and endoparasite syndromes contrasted with those observed in *Manica parasitica*.

trait	social parasite syndrome	endoparasite syndrome	*Manica parasitica*
limited geographical distribution	+	+	+
presence of multiple parasite queens in host colony (polygyny)	+	−	?
coexistence with host queen (host-queen tolerance of inquilines)	− (+)	+	?
loss of worker caste (in inquilines)	− (+)	−	−
reduced body size	+	+	+
reduced wings	+	+	?
reduced wing venation	+	?	?
reduced pilosity	+	−	−
smooth, shiny integument	+	+	+
integument colour altered	+	+	+
reduced antennal segments	+	?	−
elongated antennal scapes	+	+	+
reduced/modified mouthparts	+	?	−
reduced mandibular dentition	+	−	−
oval head	+	+	−
ocelli present	+	+	−
reduced thoracic sclerites in gyne	+	+	?
propodeum reduced	+	−	−
postpetiole broadened	+	+	+
swollen gaster	+	+	−

## 2. Material and methods

For a full description of materials and methods, see electronic supplementary material, file S1.

We located two populations of *M. parasitica* in the Sierra Nevada (figure 1*g*). Nests were excavated via shovel; workers and brood were transferred to nest-boxes for observation. To document interactions between *M. parasitica* and *M. bradleyi*, we recorded videos with a Sony AX53 4K Handycam.

To test the hypothesis that *M. parasitica* is an independently evolving species, we gathered multiple specimens of *M. parasitica* and *M. bradleyi* from the two populations (figure 1*g*), also sampling from the larger range of *M. bradleyi.* We extracted DNA, prepared genomic libraries, and performed targeted enrichment of ultraconserved elements (UCEs) [27]. We processed raw reads with the PHYLUCE and SWSC-EN pipelines [28,29], incorporating sequences from previous datasets [27,30]. We used two analytical approaches: concatenate and partition via IQTREE v. 2.1.2 [31], and summary coalescent analysis via ASTRAL-III [32]. Collection data are in electronic supplementary material, table S1. Raw data are on the NCBI Sequence Read Archive (BioProject PRJNA1013280).

To re-evaluate the morphological observations made by Creighton [25], we took morphometric data from *M. parasitica* and *M. bradleyi* using a Leica M205C microscope equipped with a Leica DFC450 digital camera and the Leica Application Suite v.4.5 (electronic supplementary material, file S1, figure S1 and table S3). Our morphometric dataset included 82 specimens (*M. parasitica*, *n* = 40; *M. bradleyi*, *n* = 42) from both populations. Raw data and scripts are available from the Dryad Digital Repository: https://doi.org/10.5061/dryad.9s4mw6mnz [33].

Because we observed no reproductives of *M. parasitica* nor *M. bradleyi* during excavations, we dissected workers of both species to determine reproductive status, following Dolezal & Brent [34]. To compare the internal anatomy of *M. parasitica* and *M. bradleyi*, we scanned three specimens each using synchrotron X-ray micro-computed tomography (microCT), processed the tomograms by segmenting every 10^th^ slice with Slicer v. 5.0.3 [35], and performed semi-automated segmentation with Biomedisa [36]. Original scans and segmentations also on the Dryad Digital Repository [33].

Metacestodes (larval cestodes) extracted from ants were fixed in 95% ethanol. Specimens were mounted on microscope slides in Berlese's medium [37]. They were examined and photographed using a Zeiss Axio Imager M2 light microscope.

## 3. Results

**Figure 2.**
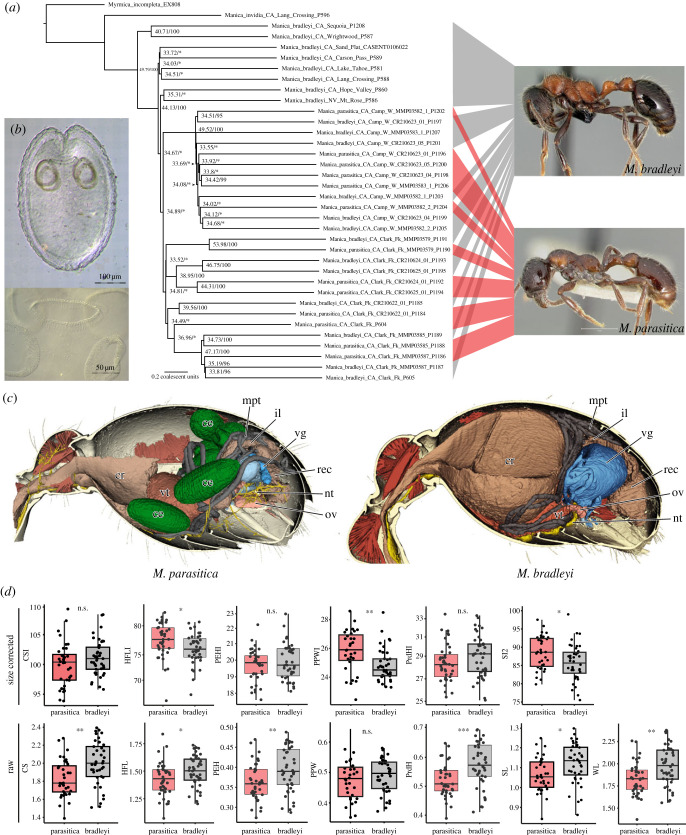
Micromorphology, phylogeny and morphometrics of *Manica parasitica* and *Manica bradleyi*. (*a*) Summary coalescent phylogeny of *Manica* inferred with ASTRAL III from UCE sequence data. (*b*) Cysticercoids of *Raillietina* (*sensu lato*) sp. from *M. parasitica*, mounted in Berlese's medium. Top: whole view. Bottom: detail of the anterior part demonstrating the armament of the rostellum and the suckers. (*c*) Cross-section of microCT reconstruction showing the *in situ* positions of metacestodes within the gaster of a *M. parasitica* worker compared with *M. bradleyi*: *ce* metacestodes; *cr* crop; *il* ileum; *mpt* Malpighian tubules; *nt* nerve tissue; *ov* ovary; *rec* rectum; *vg* venom gland; *vt* ventriculus. (*d*) Results of morphometric analysis of traits highlighted by Creighton [25]. Top row: size corrected morphometric data. *CSI* cephalic size index, *HFLI* hind femur length index, *PEHI* petiole height index, *PPWI* postpetiole width index, *PrdHI* propodeum height index, *SI2* scape index 2. Bottom row: raw data. *CS* cephalic size, *HFL* hind femur length, *PEH* petiole height, *PPW* postpetiole width, *PrdH* propodeum height, *SL* scape length, *WL* Weber's length. Significance was evaluated with a *t*-test with a threshold of 0.05.

In this study, we located two populations of *Manica parasitica*, a putative social parasite of *M. bradleyi*, in the Sierra Nevada. To test if *M. parasitica* is an independently evolving species, we inferred a phylogeny for our target taxa (*n* = 34) using broad sampling (see electronic supplementary material table S4 for sequencing statistics). We recovered a statistically well-supported clade consisting of *M. bradleyi* and *M. parasitica,* which were extensively interdigitated (figure 2*a*; electronic supplementary material, file S3: figure S1). Additionally, we recovered population structure with individuals of *M. parasitica* and *M. bradleyi* generally clustering together by population.

To evaluate the diagnostic, species-specific characters proposed by Creighton [25], we collected morphometric data. We found that *M. parasitica* is significantly smaller than *M. bradleyi* (figure 2*d*: WL), and *M. parasitica* differs significantly from *M. bradleyi* in multiple measurements (electronic supplementary material, file S3: figure S2 for full results). However, when corrected for size, many of these differences became insignificant (figure 2*d*: PEHI, PrdHI). Instead, the postpetiole was significantly broader, and the antennal scapes were significantly longer in *M. parasitica* versus *M. bradleyi,* both of which have been proposed as morphological traits similarly affected by the social parasite and endoparasite syndromes. Additionally, hind femur length was significantly longer in *M. parasitica* versus *M. bradleyi* (figure 2*d*: HFLI and SI2). Significant differences in hind femur length and antennal scape length, regardless of size correction, suggest differential effects of parasite infection on the development of the head, thorax and abdomen versus extremities, which remain less affected in parasitized individuals.

We did not recover reproductives of either species from excavations. We dissected workers of both species to determine reproductive status. We found no evidence of reproductive activity in *M. parasitica* or *M. bradleyi* workers, but the gaster of every *M. parasitica* worker (*n* = 32) contained at least one, and up to 31 metacestodes. Workers of *M. bradleyi* (*n* = 33) contained none. From our microCT reconstructions, we conclude that the metacestodes are localized in the haemocoel, without any apparent disruption to major organ systems (figure 2*c*; electronic supplementary material, table S2), although the venom gland of *M. parasitica* appeared atrophied compared to *M. bradleyi* (electronic supplementary material, file S3: figure S3).

Examination of the metacestodes revealed a pattern of body organization corresponding to cysticercoids [38] with a fully developed scolex (identical to that of adults) retracted into a solid cyst (figure 2*b*, top). They possess a rostellum armed with 194–198 hooks arranged in two regular rows in a simple circle (figure 2*b*, bottom). Hooks are shaped specifically (hammer-shaped or T-shaped), with short blade, short handle and elongate guard. Hook length measured along the axis of the guard is 14–16 μm. Suckers are armed 14–16 diagonal rows of spines situated peripherally; spines 12–13 μm long (figure 2*b*, bottom). The shape and arrangement of the rostellar hooks, in combination with armed suckers, identify this species as a member of the genus *Raillietina* Fuhrmann, 1920 (Cestoda: Davaineidae).

Following morphometric analysis, we compared *M. parasitica* against the endo- and social parasite syndromes (table 1). Although some characters of the endoparasite syndrome remain unknown, *M. parasitica* overlaps with both syndromes, e.g. reduced body size, limited geographical distribution, smooth integument, elongated antennal scapes, altered integument colour and broadened postpetiole (figure 2*d*; electronic supplementary material, table S3). However, more overlap with the endoparasite syndrome is apparent: the worker caste is not lost, and pilosity, mandibular dentition and propodeum are not reduced. *Manica parasitica* also exhibits some characteristics that defy the endoparasite syndrome, i.e. the head is not ovular, ocelli are absent and the gaster is not swollen, despite carrying a sometimes-heavy load of parasites.

## 4. Discussion

Above, we tested the hypothesis that *Manica parasitica* is an independently evolving social parasite species by (i) extensively sampling two localities, (ii) inferring the phylogeny of *M. parasitica* and *M. bradleyi* using dense population sampling, (iii) evaluating morphological and life-history characters, and (iv) using the combined evidence to evaluate *M. parasitica* in the context of the social parasite and endoparasite syndromes.

Because *M. parasitica* has an unusual combination of characters that variously overlap with (or defy) the endo- and social parasite syndromes (table 1), we initially gave credulity to Creighton's hypothesis that *M. parasitica* is a social parasite. Therefore, we had initiated the phylogenetic analysis before we realized that *M. parasitica* was infected with cestodes. Our phylogeny is consistent with the result that *M. parasitica* represents cestode-infected *M. bradleyi* workers instead of an independently evolving species. *Manica parasitica* and *M. bradleyi* samples are interdigitated within a single clade, rendering each other polyphyletic (figure 2*a*). Previous studies have identified paraphyletic host groups, with social parasite species nested within the host clade. However, in those studies, parasite taxa were monophyletic, and host paraphyly was interpreted as a signature of incomplete lineage sorting following recent speciation [39–41]. By contrast, the interdigitated pattern of *M. bradleyi* and *M. parasitica* suggests close relatedness between individuals and the absence of genetic divergence.

The morphological alterations of *M. parasitica* are apparently caused by infection with metacestodes. Remarkably, we found that a single metacestode was sufficient to induce the full suite of morphological changes in an individual (9 of 40 dissections; electronic supplementary material, table 2). We have identified the cestode genus as *Raillietina* (*sensu lato*), a cosmopolitan genus consisting of approximately 290 species that uses insects of different orders (rarely, gastropods) as intermediate hosts and non-aquatic bird and mammal species as definitive hosts [42–44]. Four subgenera have been erected [42,43] based on two binary characters of the adult morphology (genital pores unilateral/alternating; uterine capsules with a single egg/multiple eggs), which are sometimes recognized as the full genera *Raillietina* (*sensu sricto*), *Fuhrmanneta* Stiles & Orleman, 1926, *Paroniella* Fuhrmann, 1920 and *Skjabinia* Fuhrmann, 1920 [44,45]. However, these are species assemblages formed to facilitate the identification of adult cestodes rather than monophyletic groups. Of the genus *Raillietina* (*sensu lato*), there are at least 21 species recorded from North America [43]; their definitive hosts are birds of the family Phasianidae, Odontophoridae, Picidae, Icteridae and Columbidae as well as small mammals (rodents, rabbits and hares). Exact species identification of the metacestode in this study may continue with comparative studies of the scolex armaments of the known North-American *Raillietina* spp.

We remain uncertain of the details of parasite transmission, but speculate that *M. bradleyi* collects bird faeces containing cestode eggs while foraging and feeds these to its larvae via trophallaxis, which has been observed in other myrmecine ants [46]. Development of infected individuals is likely disrupted by nutrient deficiency, potentially causing the morphological modifications observed in *M. parasitica*.

While we did not conduct a quantitative analysis of behaviour in this study, we confirmed Wheeler & Wheeler's [26] observation of *M. parasitica*'s unusual gait via casual observations of multiple individuals, finding that infected workers move slowly and often fall when scaling small objects. In both populations, we observed *M. parasitica* using the same nest entrances as *M. bradleyi* (figure 1*h*). Workers of *M. parasitica* interacted normally with *M. bradleyi* nest-mates, accepting grooming from uninfected *M. bradleyi* workers (electronic supplementary material, file S2). *Manica parasitica* workers often shake as they rest or groom (electronic supplementary material, file S2). Endoparasites are known to manipulate the behaviour of their hosts, and some ‘adaptive manipulations’ are interpreted as extended phenotypes of the parasite, increasing parasite transmission [47]. The gait of *M. parasitica* may be an adaptive manipulation by the parasite, making workers easier prey for foraging birds. Alternatively, the gait of *M. parasitica* workers could be a pathological reaction or an adaptive host response [48]. The potential adaptive significance of this behaviour must be determined experimentally.

We remain uncertain of the significance, if any, of the reduced integument sculpture and altered coloration of parasitized individuals. Reduced sculpturing may be a product of thinner cuticle, which may be caused by the parasite reappropriating resources during larval development. Darker coloration of parasitized individuals has also been observed in *Myrmica* infected with davaineid cestodes [49]*.* Dark cuticle could be caused by an immune response to the parasite infection because melanization is one of many responses that arthropod immune systems use to combat infections [50,51]. If infection occurs before pupation, melanin may affect the adult tanning process, resulting in darker individuals.

Considering the combined evidence, we conclude that *M. parasitica* represents cestode-infected *M. bradleyi* workers rather than an interspecific social parasite of *M. bradleyi*. Accordingly, we propose the taxonomic synonymy of *M. parasitica* (Creighton, 1934) (**syn. n.**) under *M. bradleyi* (Wheeler, 1909). We would like to reiterate the findings of Csősz *et al*. [19] by emphasizing that, whenever possible, multiple lines of evidence should be used when describing new taxa. This especially applies to putative social parasite species given their rarity and the overlaps between the social parasite and endoparasite syndromes (table 1). While there are multiple hypotheses for the adaptive significance of traits listed in table 1 for the endo- and social parasite syndromes, to our knowledge few have been formally tested. Future studies are needed to shed light on the identity and life cycle of *Raillietina* cestodes infecting *M. bradleyi*, and to test the adaptive significance of the morphological and behavioural modifications exhibited by ants infected with endoparasites, as well as by ant social parasites.

## Data Availability

The sequence reads generated in this study are available from the NCBI Sequence Read Archive (BioProject PRJNA1013280). The data and scripts that support the findings of this study are available from the Dryad Digital Repository: https://doi.org/10.5061/dryad.9s4mw6mnz [33]. Supplementary material is available online [52].
